# Impact of ELKa, the Electronic Device for Prandial Insulin Dose Calculation, on Metabolic Control in Children and Adolescents with Type 1 Diabetes Mellitus: A Randomized Controlled Trial

**DOI:** 10.1155/2017/1708148

**Published:** 2017-01-23

**Authors:** Agnieszka Kowalska, Katarzyna Piechowiak, Anna Ramotowska, Agnieszka Szypowska

**Affiliations:** Pediatric Hospital, Department of Pediatrics and Pediatric Diabetes, Warszawski Uniwersytet Medyczny, Ul. Żwirki i Wigury 63A, 02-091 Warsaw, Poland

## Abstract

*Background*. The ELKa system is composed of computer software, with a database of nutrients, and a dedicated USB kitchen scale. It was designed to automatize the everyday calculations of food exchanges and prandial insulin doses.* Aim.* To investigate the influence of the ELKa on metabolic control in children with type 1 diabetes mellitus (T1DM).* Methods*. A randomized, parallel, open-label clinical trial involved 106 patients aged <18 years with T1DM, HbA_1C_ ≤ 10%, undergoing intensive insulin therapy, allocated to the intervention group, who used the ELKa (*n* = 53), or the control group (*n* = 53), who used conventional calculation methods.* Results*. After the 26-week follow-up, the intention-to-treat analysis showed no differences to all endpoints. In per protocol analysis, 22/53 (41.5%) patients reporting ELKa usage for >50% of meals achieved lower HbA_1C_ levels (*P* = 0.002), lower basal insulin amounts (*P* = 0.049), and lower intrasubject standard deviation of blood glucose levels (*P* = 0.023) in comparison with the control. Moreover, in the intervention group, significant reduction of HbA_1C_ level, by 0.55% point (*P* = 0.002), was noted. No intergroup differences were found in the hypoglycemic episodes, BMI-SDS, bolus insulin dosage, and total daily insulin dosage.* Conclusions*. The ELKa system improves metabolic control in children with T1DM under regular usage. The trial is registered at ClinicalTrials.gov, number NCT02194517.

## 1. Introduction

For patients remaining under intensive insulin therapy, prandial insulin dose calculation based on the establishment of the contents of carbohydrates (CHO) in food is the key to optimizing postprandial glycemia levels [1–3]. Additionally, calculating fat and protein units helps to improve postprandial excursions caused by high in fat and protein meals [4, 5]. For many patients or caregivers, the conventional method of calculation, which requires the food to be weighed and tables with nutritional data to be consulted, is complex, time-consuming, and difficult to perform routinely. In Bishop et al.'s study, only 23% of the adolescents with type 1 diabetes (T1DM) estimated CHO content within 10 g of the true amount per day [6].

**Figure 1:**
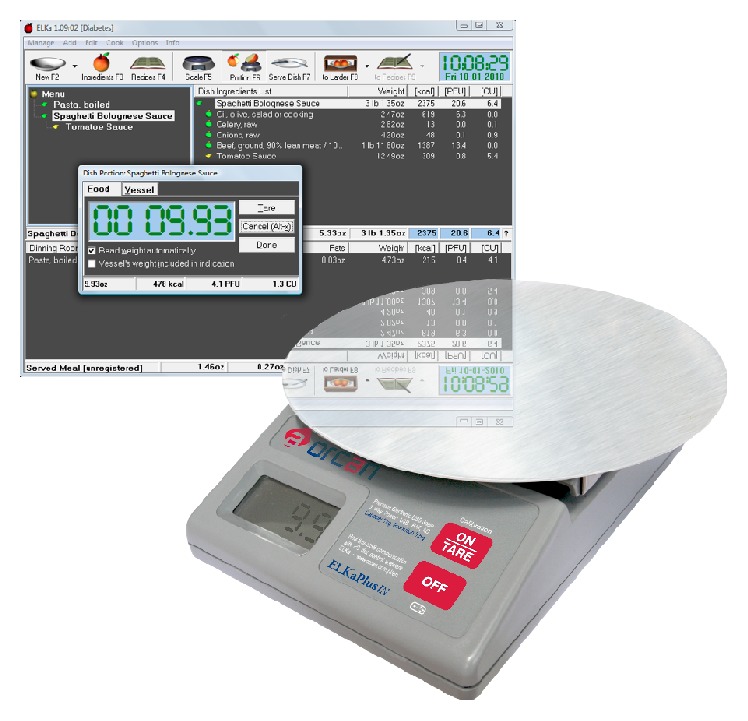
The ELKa toolset.

Considering a rapid development of personal computers, it has become tempting to incorporate them into dietary management. A parent of our former patient developed the ELKa system (Orcan, Warsaw, Poland) to simplify and shorten everyday calculations of CHO units and fat-protein units of homemade food with a constant precision of the results [7]. The toolset consists of an ELKa Windows® application, distributed as freeware in both Polish and English versions, and an ELKaPlus digital kitchen scale (Figure 1). During the meal preparation, the user creates or reloads the list of ingredients in the software basing on an incorporated ample food database and weighs them on the ELKaPlus kitchen scale. The scale transmits the weight of the ingredients in real-time to the computer via a USB port. As a result, the user receives automatically calculated data with the number of calories, CHO, fat, and protein content in the meal. The program is able to perform calculations for any complex meal taking into account weight changes after thermal processing.

Few studies have been performed to evaluate other computer/smartphone applications facilitating food calculations and dedicated for people with diabetes [8–10]. To our best knowledge, the only software with available trial results that seems comparable to the ELKa system is Diabetics [11]. Nevertheless, the requirement of Diabetics to manually enter all meal components may be considered too time-consuming for many patients. The ELKa innovative solution differs from other systems by using a real-time transmission of the ingredients' weight onto the computer. This feature allows users to calculate food exchanges without having to manually enter each weighing result. We hypothesize that the features offered by the ELKa toolset may have positive influence on patients' compliance and improve diabetes control in a long-term follow-up. Our clinical experience shows that ELKa users achieved good glycemic control; however, no trials assessing the ELKa influence on metabolic control had been performed prior to this study.

The aim of the study was to evaluate the efficacy of the ELKa toolset in the improvement of metabolic control in children with T1DM.

## 2. Subjects and Methods

### 2.1. Trial Design and Randomization Details

A randomized, controlled, unblinded clinical trial was conducted in 106 pediatric patients with type 1 diabetes, assigned to one of the two parallel groups with a 1 : 1 allocation ratio. An independent party created a computer-generated, simple randomization list. To conceal the number sequence, the list was kept by a coworker not involved in the study. After the completion of the subject's all baseline assessments, the allocation was unveiled to the enrolling investigator on demand. Because of the type of the intervention, blinding was not possible.

### 2.2. Participants

The inclusion criteria were age less than 18 years, T1DM recognized at least 1 year before, HbA_1C_ level ≤ 10% (86 mmol/mol), undergoing intensive insulin therapy, the patient's kitchen arrangement providing enough space for the toolset, and the patient disposing of a computer meeting the ELKa system requirements. Patients who were excluded met at least one of the following criteria: nutritional disorders, celiac disease recognized less than 4 months before the enrolment, a preceding experience with the software, and expecting 21 or more consecutive days pausing in the system usage.

Written, informed consent from patients aged 16 years or more and all parents/caregivers has been obtained. This study has been performed according to the Declaration of Helsinki and has been approved by the Bioethics Committee at the Medical University of Warsaw (KB17/2012).

The trial was conducted in the Outpatient Clinic of the Department of Pediatrics, at the Medical University of Warsaw between April 2013 and October 2014. Participants were assessed and recruited on their routine follow-up appointments in the Outpatient Clinic. All subjects were treated with insulin pumps and none of them used continuous glucose monitoring. All outpatients and/or their caregivers were previously trained in intensive insulin therapy in the Department of Pediatrics. As part of standard education, the patients and their parents/caregivers learned about the calculation of CHO portions (1 CHO portion = 10 g of CHO after fiber subtraction), the calculation of fat and protein units using approved caloric and nutrients tables [12] (1 fat-protein unit = 100 kcal from fat and/or protein [5]), the calculation of prandial insulin dose with the insulin-to-carbohydrate ratio, insulin-to-fat-protein ratio and correction factor, the usage of different types of boluses, the basal insulin modifications, and the principles of a healthy diet based on natural, low-processed ingredients with a consideration of glycemic index and load.

### 2.3. Procedure

At the trial entry, the eligible patients were randomly assigned to one of the two groups: the intervention group, given the ELKa toolset, or the control group. In every group, the patients and/or the caregivers were involved in everyday meal preparations, received a short, recall training about the rules of exchange calculation and the proper insulin dosage, and filled a questionnaire concerning computer skills and sociological data. Additionally, the intervention group was trained in the ELKa toolset usage. To minimize the risk of variability in teaching, only two persons performed the training interchangeably and according to the previously agreed program: the developer of the software and a skilled pediatrician. The patients in the control group were requested to continue their prandial insulin dose calculations based on their common habits.

Additionally to the baseline visit, there were two follow-up visits: one after the 13th week and one after the 26th week, scheduled simultaneously with routine appointments with the diabetologist in the Outpatient Clinic to minimize the risk of absence. During all 3 appointments, HbA_1C_ levels, body weight, and height were measured, and frequency of severe hypoglycemia events was assessed.

AccuChek 360 software (Roche Diabetes Care, Indianapolis, IN, USA) and CareLink Pro Therapy Management Software (Medtronic, Minneapolis, MN, USA) were used to download data from insulin pumps. All data concerning insulin dosage and bolus types from 14 days preceding the doctor's appointment were analyzed. Blood glucose levels results derived from the self-monitoring of blood glucose and were used for the assessment of mean glycemic values and hypoglycemia rate. Data, gathered during a 14-day time frame preceding the first or the follow-up visits with at least 4 entries per day, were considered. AccuChek 360 software was used for reading *n* = 51 sets of glucometers' data. CareLink software was used for reading *n* = 147 sets of data. Remaining glucose meters were read with the use of other dedicated software (Gluco Contro v. 1.5.2.2650 (Bayer, Leverkusen, Germany) for the *n* = 7; FreeStyle Auto-Assist v2.0 (Abbott Diabetes Care, Alameda, CA, USA) for *n* = 3; OneTouch® Zoom® Pro Diabetes Management Software v5.1.1, (LifeScan, Chesterbrook, PA, USA) for *n* = 15 datasets downloads) or hand copied if electronic data collection was not possible.

The intervention group at follow-up visits was asked to determine the frequency of the toolset usage, the satisfaction, and the wish to continue.

### 2.4. Outcomes

A change in HbA_1C_ after 26 weeks of observation was the primary endpoint.

Secondary endpoints were HbA_1C_ after 13 weeks, ELKa usage frequency, differences in the total daily insulin doses (TDD), the daily doses of basal insulin, the total daily doses of bolus insulin, basal as % of TDD, the number of different types of boluses, and the changes in Body Mass Index-Standard Deviation Score (BMI-SDS). Furthermore, other secondary endpoints were data concerning glycemic values: the daily mean, the diurnal mean (measures between 07:00 AM and 09:59 PM) and the nocturnal mean (10:00 PM–06.59 AM) of blood glucose levels, and glycemic variability assessed by analyzing standard deviation (SD) of the given set of blood glucose levels results.

HbA_1C_ was measured using a high-pressure liquid chromatography method with normal range 4.1–6.4% (21–46 mmol/mol) for patients without diabetes.

Severe hypoglycemic events were defined as hypoglycemia with the presence of seizure and/or unconsciousness, requiring administration of glucagon or intravenous glucose infusion. The number of episodes was established on the basis of the patients' medical history and interview with researcher performed on each follow-up visit. Hypoglycemia, defined as glycemia below 70 mg/dL (<3.9 mmol/L) and, separately, glycemia below 50 mg/dL (2.5 mmol/L) were also assessed on the basis of the data from self-monitoring of blood glucose and were expressed as a number of episodes in a 2-week period per patient per 24 h.

Compliance was assessed on an interview with caregivers supported by a questionnaire and described as the percentage of meals prepared with the help of the ELKa toolset. BMI-SDS calculations were performed by using WHO Anthro Plus software (v.1.0.4, WHO, Geneva, Switzerland) based on WHO standards (birth to 60 months) and WHO reference 2007 (61 months to 19 years).

### 2.5. Statistical Methods

Statistical analyses were performed using StatsDirect v.2.8.0 (StatsDirect Ltd., Altrincham, England, UK). To detect a difference of 0.5% point (5.5 mmol/mol) in HbA_1C_ levels with *α* = 0.05 and a power of 80%, a minimum number of 52 participants per group were necessary. The study was performed on our chronic patients and follow-up visits were planned during routine appointments with a diabetologist in the Outpatient Clinic. Because we did not expect a significant dropout rate, the study group was increased by 2 patients only.

Descriptive statistics was used for patients' characteristic. D'Agostino and Pearson and Shapiro–Wilk normality tests were used to confirm the normality of data distribution. The primary analysis was performed by the intention-to-treat (ITT) approach. The results were analyzed using the available case analysis. We performed a per protocol (PP) analysis on patients who received the allocated intervention with a declared ELKa system usage for minimum 50% of meals. Student's *t*-test was applied for intergroup and intragroup comparisons of HbA_1C_ and secondary outcomes comparisons; alternatively the Mann–Whitney *U* test and Fisher's exact test for nonparametric and ordinal-scaled data were applied. Wilcoxon matched-pairs signed-rank test was also used for intragroup comparisons of changes in HbA_1C_ between visits. The blood glucose variability was assessed by analyzing SD of the set of glycemic measurements (separately daily, diurnal, and nocturnal).

Results are shown as a mean with SD. Differences between the study groups were considered significant when *P* value was <0.05.

## 3. Results

### 3.1. Participant Flow

**Figure 2:**
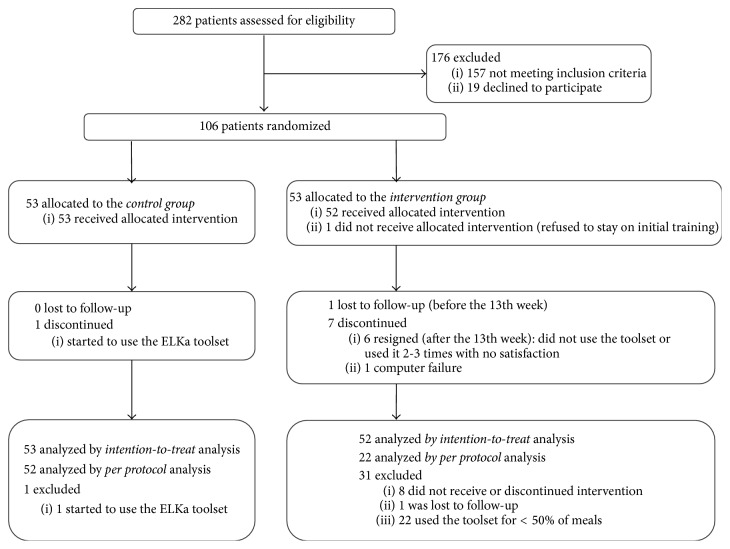
Participants' passage through the study.

Between April 2013 and April 2014 282 patients were assessed for eligibility and 106 patients were randomized to the intervention group (*n* = 53) or to the control group (*n* = 53).  Figure 2 shows the participants' passage through the study. Main exclusion reasons were HbA_1C_ level >10% (86 mmol/mol), a previous experience with the device, and not enough space in the kitchen for the toolset. Patients, who refused to participate, preferred to estimate the CHO, fat, and protein content and were not interested in changing the method. Regarding the baseline characteristic, groups were well balanced with the exception of age, as it is summarized in Table 1. Three patients were found to meet postrandomization exclusion criteria: two with newly diagnosed celiac disease after the 6th and the 8th week of the trial and one patient who learned to be pregnant after the 16th week of the trial. Nevertheless, they remained included in the ITT and PP analyses. Additionally, none of the patients reported continuous glucose monitoring system usage during the study period.

### 3.2. HbA_1C_

In the ITT analysis, we found no significant differences in HbA_1C_ levels between groups after 13 (*P* = 0.085) and 26 weeks of observation (*P* = 0.156) (Table 2). The PP population significantly differed in HbA_1C_ result from the control group after 26 weeks of the toolset usage (*P* = 0.002). The difference after 13 weeks was noticeable, but statistically insignificant (*P* = 0.149) (Table 3).

Regarding changes in HbA_1C_ over time, reduction in HbA_1C_ levels was observed within the intervention group only. In ITT analysis, the results were statistically significant after 3 months but not after 6 months; in PP analysis, the HbA_1C_ levels were lowering throughout the whole study, with a difference close to statistical significance after 3 months (−0.45% point; −4.9 mmol/mol; *P* = 0.054) and a significant difference of −0.55% point (6 mmol/mol; *P* = 0.002) after 6 months. No significant HbA_1C_ reduction was found within the control group either in ITT or PP analysis (Table 4).

### 3.3. Insulin Dosage and Bolus Types

In the ITT analysis, regarding available cases, basal insulin dosage significantly differed between groups after 13 weeks of observation (*P* = 0.03), and these differences were close to statistical significance after 26 weeks (*P* = 0.06). Similar results were observed for basal as % of TDD (13 weeks: *P* = 0.027, 26 weeks *P* = 0.067) (Table 2). In the PP analysis, basal insulin amounts were lower in the intervention group after 13 weeks (*P* = 0.005) and 26 weeks (*P* = 0.049), and differences in basal as % of TDD were significant or close to statistical significance (13 weeks: *P* = 0.034, 26 weeks *P* = 0.057) (Table 3). With regard to total daily doses of bolus insulin and TDD, no significant differences were observed between the groups either in the ITT or in the PP analyses (Tables 2 and 3).

The mean number of all and particular types of boluses per 24 h as well as proportions between them did not change throughout the study (see Supplementary Table  1 in the Supplementary Material available online at https://doi.org/10.1155/2017/1708148).

### 3.4. Blood Glucose Levels and Stability

The mean blood glucose levels did not differ significantly between groups except for nocturnal glycemic values after 13 weeks (*P* = 0.016) in the ITT analysis and daily (*P* = 0.041) and nocturnal (*P* = 0.009) glycemic values after 13 weeks in the PP analysis. Nevertheless, SD, as a measure of the intrasubject instability of glucose levels, achieved significantly lower values in the intervention group in the PP approach (except for diurnal values after 13 weeks), despite the lack of differences shown in the ITT analysis (Tables 2 and 3).

### 3.5. Hypoglycemia Episodes

Severe hypoglycemic episodes were not present in any group during the study period. Additionally no significant differences between groups regarding glycemia levels <70 mg/dL, and separately <50 mg/dL, were found (Supplementary Table  1).

### 3.6. Frequency and Satisfaction of the Toolset Usage

After 26 weeks, the ITT analysis provided the following results: 22/53 (41.5%) patients allocated to the intervention group reported ELKa usage for more than 50% of meals, with 7/53 (13,2%) for more than 85% of meals and 15/53 (28.3%) for 〈51–85%〉 of meals. 16/53 (30.2%) declared the toolset usage for 11–50% of meals. 15/53 (28.3%) patients used the toolset for less than 11% of meals, of whom 6/53 (11.3%) patients admitted to occasional toolset usage (11.3%), 1/53 (1.9%) was willing to use the toolset but with computer failure, 1/53 (1.9%) was lost to follow-up, 1/53 (1.9%) did not receive the allocated intervention, and 6/53 (11.3%) resigned from the trial. Furthermore, from the intervention group, 33/52 (63.5%) patients were satisfied and were willing to continue using the ELKa toolset.

No intergroup differences in BMI-SDS were observed (Supplementary Table  1).

No adverse events or side effects were reported.

### 3.7. Additional Analyses

Post hoc comparisons were performed after excluding three patients who were found to meet postrandomization exclusion criteria: two patients with celiac disease diagnosed on the 6th or 8th week and a patient who was pregnant on the 26th week of the follow-up. The results did not differ significantly from those presented in Table 2.

## 4. Discussion

This prospective, open-label, randomized, controlled trial shows, in the ITT analysis, that the ELKa system usage has no longitudinal influence on metabolic control in children with T1DM treated with an insulin pump. The assessment was based on differences in HbA_1C_ levels between groups during a 6-month observation. However, in the PP approach ELKa active users significantly differed, regarding the primary endpoint, from the control group. The improvement in HbA_1C_ results was not associated with the increased rate of hypoglycemic episodes. Moreover, basal insulin amount and blood glucose levels variability were lower in the intervention group on both follow-up visits.

Similarly to our study, Blazik and Pańkowska [13] did not observe in the ITT any improvement in HbA_1C_ values after a 3-month observation; nevertheless, in that study both a small number of patients (*n* = 48) and a short time frame might have influenced the result.

In PP analysis, after six months of observation, we noticed that the patients in the intervention group, who regularly used ELKa, not only significantly differed from the control group in the HbA_1C_ result, but also achieved a significant intragroup reduction of HbA_1C_ level, by 0.55% point (6 mmol/mol). Because of the initial HbA_1C_ level being low (approximately 7.5% (58 mmol/mol)), this improvement would have seemed difficult to achieve.

We hypothesize that the impressive HbA_1C_ result is a consequence of an improvement in food calculation accuracy along with having not only the CHO but also the fat and protein units counted with precision [14]. While a 10 g margin of error has no major consequences for postprandial glycemia in children aged 9.5 years or more [15], a lack of precision in the CHO calculations with 20 g error leads to significant disturbances in postprandial glycemia, including an increased risk of hypoglycemia [16]. Furthermore, we consider the added value of the patients' learning: using the ELKa toolset on daily basis, patients receive information of the exact amount of CHO, fat, and protein on their plate, along with the real look of the meal. They gain an experience probably far better than after a training with 2D food photography, as it was proposed by Gandolfo et al. [17], and improve their estimation abilities for the moments when they cannot use the toolset, such as while eating out unlabeled food or large meals, which are usually more challenging to estimate [18]. Moreover, the appropriate calculation of calories of the meal may prevent weight gain, hence the importance of fat and protein amount calculations. We did not observe any changes in the BMI-SDS. The results observed are in accordance with studies on the introduction of advanced CHO content calculation, which state that it appeared to have variable influence on the BMI, with minor or no changes, as Schmidt et al. summarized it in the review [19]. Significant, but transient decrease of HbA_1C_ levels observed in the experimental group in the ITT analysis may be explained by the run-in effect of the trial participation.

Foregoing arguments may also be applied to explain temporary changes in proportions between basal and boluses insulin in the experimental group. Regarding the PP analysis, we hypothesize that differences in basal doses in the experimental group without changes in the TDD are a result of a better adjustment of bolus insulin. We observe, in clinical practice, that some patients overdose basal insulin in order to partially compensate for the prandial requirement, as it was also hypothesized in Holterhus et al. study [20]. This conduct may increase the risk of hypoglycemia when a meal is omitted.

The regular use of the ELKa toolset during the trial period resulted in significant improvement in day-to-day stability of blood glucose levels. We interpret this observation as a consequence of an increased precision in insulin dosage. Blazik and Pańkowska report similar findings, regarding the mean glycemic values as well as SD, but state no differences in basal and total insulin doses [13].

In our study, after the 6-month trial, 22/53 (41.5%) patients allocated to the intervention group reported ELKa usage for more than 50% of meals. The vast majority of rejections were observed during the first 3 months of the trial. It may be explained by difficulty of the software itself, but, evenly, by the difficulties in changing the patients' diabetes management style. We know from clinical practice how difficult it is to motivate patients to calculate the food when weighing the products is necessary. Because of the lack of sufficient data, it is hard to compare the frequency of the ELKa usage against the compliance with the classic method of calculation. Laurenzi et al. report that, despite recommendation, only 20 (or 22)/30 (66–73%) adults with T1DM treated with insulin pumps performed CHO calculations, with significant differences in HbA_1C_ results solely in the PP analysis [21]. It is worth noticing that such a milestone as the introduction of CHO calculations appeared to have had a variable influence on patients' HbA_1C_, according to results of systematic review, performed by Schmidt et al. [19]. Moreover analyzed population included *n* = 51 (48.1%) of adolescents, who were > 12 years old at the study onset. From our clinical experience, children in this age are not too focused on their chronic illness and are not too willing to cooperate with their caregivers in order to manage their diabetes. Therefore, the fact that after 6 months more than 40% of families remained active ELKa users may be interpreted as quite a good result.

### 4.1. Trial Strengths and Limitations

We worked with a precisely diagnosed, homogenous group of patients remaining under constant care of our Outpatient Clinic; therefore, the lost-to-follow-up rate was 1/106 (0.94%). We used a proper method of allocation, with its concealment. Six months of follow-up seems to have eliminated the run-in-effect in the study. The analysis was performed using the ITT approach with no exclusions except for missing data.

One of potential limitations was the lack of blinding, which resulted from the nature of the intervention and could not have been avoided. Another limitation was the difference in age between the study groups: the patients in the control group were older, but the age difference was small; therefore we do not believe that this has put the study results into question. As the ELKa software has no automatic user's activity recording, no impartial method for adherence measurement was possible to apply. To obtain reliable data, we were performing structured interviews with the patients and the caregivers, based on predefined distractors in a provided questionnaire, precisely describing possible variants.

## 5. Conclusions

The results of our trial prove that ELKa usage provides its users with a clinically important improvement in metabolic control as well as it helps to stabilize day-to-day insulin dosage. Our study concerned a broad group of pediatric patients with type 1 diabetes who were asked to use the software as frequently as they managed to, but without special forcing, in order to possibly imitate normal conditions. Therefore we assume that the general population of patients may benefit from using the toolset.

Regarding the compliance and the PP analysis results, we believe that the ELKa system will be a useful tool for well-motivated patients who require and wish to improve on their food calculation. The tool may especially prove useful to patients who, despite being already well metabolically controlled and performing everyday calculations using the standard method, still struggle to reduce further their HbA_1C_ levels and achieve normoglycemia.

## Supplementary Material

The results concerning mean number of all and particular types of boluses per 24 h, proportions between them, hypoglycemic events frequency and BMI–SDS values are included in the Supplementary Table 1. Regarding all presented endpoints, neither intention-to-treat analysis nor per protocol analysis revealed significant differences between the groups.

## Figures and Tables

**Table 1:** Baseline characteristics of the study population.

	ELKa (*n* = 53)	Control (*n* = 53)	*P* value
Men (*n* (%))	20 (37.7%)	22 (41.5%)	0.843
Age at inclusion [years]	9.8 (4.3)	12.1 (3.7)	**0.006**
Living in town > 20,000 habitants (*n* (%))	36 (67.9%)	29 (54.7%)	0.231
Diabetes duration [years]	4.52 (2.7)	5.4 (3.5)	0.266
HbA_1C_ [%]	7.6 (1)	7.4 (1)	0.283
Treatment with CSII (*n* (%))	53 (100%)	53 (100%)	1
TDD [IU/kg/24 h]	0.79 (0.15)	0.81 (0.2)	0.479
Basal insulin^a^ [IU/kg/24 h]	0.23 (0.1)	0.3 (0.1)	0.429
BMI-SDS	0.8 (0.8)	0.6 (1)	0.351
SMBG^a^ [*n*/24 h]	7.5 (1.8)	7.4 (1.7)	0.826
Family members involved in CHO/FP exchanges calculation^a^ [*n*]	2.2 (0.8)	2 (1)	0.104
Caregiver's age^a^ [years]	38.6 (7)	39.5 (6)	0.467
Caregivers who completed at least upper secondary education level^a^ (*n* (%))	47 (88.7%)	47 (88.7%)	1
Caregivers using computer for >1/week^a^ (*n* (%))	49 (92.4%)	42 (79.2%)	0.092
Caregivers having difficulties with computer usage^a^ (*n* (%))	2 (3.8%)	5 (9.4%)	0.437

Values are mean (SD) unless stated otherwise in round brackets. Units are shown in square brackets. CSII: Continuous Subcutaneous Insulin Infusion; TDD: total daily dose of insulin; BMI–SDS: Body Mass Index-Standard Deviation Score; SMBG: self-monitoring of blood glucose; CHO: carbohydrates; FP: fat/protein.

^a^Self-reported at the baseline.

**Table 2:** Summary results of the intention-to-treat analysis.

	Group				*P* value
	ELKa		Control		
	*n*	Mean (SD)	*n*	Mean (SD)	
HbA_1C_ [%]					
Baseline	53	7.6 (1)	53	7.4 (1)	0.283
13 w	46	7.2 (1.1)	51	7.6 (1.1)	0.085
26 w	52	7.4 (1.2)	53	7.6 (0.8)	0.156
Insulin dosage^a^					
Basal insulin [IU/kg/24 h]					
Baseline	47	0.29 (0.14)	53	0.29 (0.11)	0.823
13 w	39	0.28 (0.14)	44	0.31 (0.11)	**0.03**
26 w	41	0.27 (0.11)	45	0.32 (0.1)	0.06
TDBD [IU/kg/24 h]					
Baseline	47	0.5 (0.14)	53	0.52 (0.18)	0.521
13 w	39	0.53 (0.15)	44	0.5 (0.14)	0.326
26 w	41	0.53 (0.13)	45	0.5 (0.14)	0.445
TDD [IU/kg/24 h]					
Baseline	47	0.79 (0.15)	53	0.81 (0.21)	0.479
13 w	39	0.81 (0.19)	44	0.81 (0.19)	0.978
26 w	41	0.8 (0.13)	45	0.82 (0.18)	0.318
Basal as % of TDD					
Baseline	47	0.37 (0.14)	53	0.37 (0.12)	0.94
13 w	39	0.35 (0.13)	44	0.39 (0.12)	**0.027**
26 w	41	0.35 (0.13)	45	0.4 (0.11)	0.067
Mean glucose level [mg/dL]					
Daily^b^					
Baseline	36	163 (36.3)	38	170 (36.5)	0.439
13 w	34	166 (41.7)	37	176 (33)	0.137
26 w	39	164 (34.9)	35	162 (30.8)	0.821
Diurnal^c^					
Baseline	36	160 (35.2)	38	171 (43.1)	0.249
13 w	34	169 (46.9)	37	173 (35.7)	0.369
26 w	39	163 (39)	35	161 (32.9)	0.797
Nocturnal^d^					
Baseline	36	170 (58.1)	37	171 (36.5)	0.254
13 w	34	158 (34.3)	36	180 (39)	**0.016**
26 w	39	166 (35.8)	35	167 (31.9)	0.929
SD of mean glucose level					
Daily^b^					
Baseline	36	81 (26.2)	38	79.3 (25.8)	0.927
13 w	34	80.2 (25.3)	37	84.4 (14.6)	0.14
26 w	39	78.9 (23.3)	35	81.1 (20.6)	0.327
Diurnal^c^					
Baseline	36	79 (25.7)	38	79.8 (26.3)	0.897
13 w	34	79.6 (27.7)	37	82.4 (16.1)	0.597
26 w	39	77.8 (25.3)	35	82 (22)	0.223
Nocturnal^d^					
Baseline	36	74.5 (35.8)	37	74.7 (28.5)	0.587
13 w	34	74.4 (26.1)	36	81.5 (24.9)	0.239
26 w	39	75.8 (24.7)	35	76.3 (23.3)	0.525

Intention-to-treat analysis concerns all available cases.

*n*, number of patients included in analysis; w, weeks; TDBD, total daily dose of bolus insulin; TDD, total daily dose of insulin. Units are listed in square brackets in the first column.

^a^Data downloaded from insulin pumps. ^b^Data downloaded from blood glucose meters, measured over 24 h. ^c^Data downloaded from blood glucose meters, measured between 07:00 AM and 09.59 PM. ^d^Data downloaded from blood glucose meters, measured between 10:00 PM and 06:59 AM.

**Table 3:** Summary results of the per protocol analysis.

	Group				*P* value
	ELKa		Control		
	*n*	Mean (SD)	*n*	Mean (SD)	
HbA_1C_ [%]					
13 w	23	7.1 (1.1)	50	7.6 (1.1)	0.149
26 w	22	6.9 (0.8)	52	7.6 (0.8)	**0.002**
Insulin dosage^a^					
Basal insulin [IU/kg/24 h]					
13 w	18	0.24 (0.1)	43	0.31 (0.11)	**0.005**
26 w	18	0.26 (0.1)	44	0.32 (0.1)	**0.049**
TDBD [IU/kg/24 h]					
13 w	18	0.51 (0.15)	43	0.5 (0.14)	0.702
26 w	18	0.52 (0.11)	44	0.5 (0.14)	0.617
TDD [IU/kg/24 h]					
13 w	18	0.75 (0.17)	43	0.81 (0.19)	0.298
26 w	18	0.78 (0.11)	44	0.82 (0.18)	0.42
Basal as % of TDD					
13 w	18	0.33 (0.12)	43	0.39 (0.12)	**0.034**
26 w	18	0.34 (0.12)	44	0.4 (0.11)	0.057
Mean glucose level [mg/dL]					
Daily^b^					
13 w	18	156 (29.7)	37	176 (33)	**0.041**
26 w	18	156 (26.5)	35	162 (30.8)	0.456
Diurnal^c^					
13 w	18	158 (35)	37	173 (35.7)	0.141
26 w	18	155 (31)	35	161 (32.9)	0.514
Nocturnal^d^					
13 w	18	152 (26.5)	36	180 (39)	**0.009**
26 w	18	159 (26.7)	35	167 (31.9)	0.369
SD of mean glucose level					
Daily^b^					
13 w	18	73.6 (18.8)	37	84.4 (14.6)	**0.023**
26 w	18	69.2 (14.6)	35	81.1 (20.6)	**0.017**
Diurnal^c^					
13 w	18	72.8 (23.1)	37	82.4 (16.1)	0.078
26 w	18	69.4 (17.3)	35	82 (22)	**0.019**
Nocturnal^d^					
13 w	18	65.4 (20.8)	36	81.5 (24.9)	**0.022**
26 w	18	65.7 (16.5)	35	76.3 (23.3)	**0.028**

Per protocol analysis concerns patients who received allocated intervention and declared ELKa system usage for more than 50% of meals.

*n*, number of patients included in analysis; w, weeks; TDBD, total daily dose of bolus insulin; TDD, total daily dose of insulin. Units are listed in square brackets in the first column.

^a^Data downloaded from insulin pumps. ^b^Data downloaded from blood glucose meters, measured over 24 h. ^c^Data downloaded from blood glucose meters, measured between 07:00 AM and 09.59 PM. ^d^Data downloaded from blood glucose meters, measured between 10:00 PM and 06:59 AM.

**Table 4:** Intragroup change in HbA_1C_ levels.

Group	Number of pairs	HbA_1C_ [%], mean (SD)			Change	95% CI	*P* value
		Baseline	13 w	26 w			
*Intention-to-treat approach*							
ELKa	46	7.59 (1.05)	7.24 (1.07)		−0.45	(−0.64 to −0.06)	**0.011**
ELKa	52	7.58 (1.03)		7.45 (1.19)	−0.1	(−0.38 to 0.12)	0.305
Control	51	7.43 (0.99)	7.6 (1.08)		+0.1	(−0.11 to 0.44)	0.321
Control	53	7.4 (0.99)		7.57 (0.75)	+0.17	(−0.08 to 0.43)	0.178
*Per protocol approach*							
ELKa	23	7.55 (1.2)	7.1 (1.05)		−0.45	(−0.91 to 0.01)	0.054
ELKa	22	7.48 (1.09)		6.93 (0.84)	−0.55	(−0.87 to −0.23)	**0.002**
Control	50	7.41 (0.99)	7.6 (1.09)		+0.15	(−0.08 to 0.47)	0.229
Control	52	7.38 (0.99)		7.58 (0.76)	+0.2	(−0.05 to 0.46)	0.117

Change in mean HbA_1C_ values within each group over 3 and 6 months.

Intention-to-treat analysis concerns all available cases. Per protocol analysis concerns patients who received allocated intervention and declared ELKa system usage for more than 50% of meals.
