# Supplementary material for: Impact of ELKa, the Electronic Device for Prandial Insulin Dose Calculation, on Metabolic Control in Children and Adolescents with Type 1 Diabetes Mellitus: A Randomized Controlled Trial
Source: J Diabetes Res. 2017 Jan 23;2017:1708148. doi: 10.1155/2017/1708148 (PMC5292387; doi:10.1155/2017/1708148)
Supplement: Supplementary file 1 — The results concerning mean number of all and particular types of boluses per 24 h, proportions between them, hypoglycemic events frequency and BMI–SDS values are included in the Supplementary Table 1. Regarding all presented endpoints, neither intention-to-treat analysis nor per protocol analysis revealed significant differences between the groups. [file 1708148.f1.docx]

Supplementary table 1. Summary of auxiliary results.

|  |  |  | **Intention-to-treat analysis** | | | | | | **Per protocol analysis** | | |  | |
| --- | --- | --- | --- | --- | --- | --- | --- | --- | --- | --- | --- | --- | --- |
|  |  |  | ELKa group | | Control group | |  | | ELKa group | | Control group | |  |
|  |  |  | n | Mean (SD) | n | Mean (SD) | *P*-value |  | n | Mean (SD) | n | Mean (SD) | *P*-value |
| **Types of boluses** ^a^ | | |  |  |  |  |  |  |  |  |  |  |  |
|  | All types [n/24h] | Baseline | 47 | 9.42 (5.21) | 53 | 8.48 (3.22) | 0.955 |  |  |  |  |  |  |
|  |  | 13 w | 39 | 9.1 (4.48) | 44 | 8.2 (3.15) | 0.708 |  | 18 | 10.19 (5.46) | 43 | 8.22 (3.18) | 0.348 |
|  |  | 26 w | 41 | 9.25 (5.06) | 45 | 8.1 (3.1) | 0.742 |  | 18 | 11.14 (6.35) | 44 | 8.12 (3.14) | 0.134 |
|  |  |  |  |  |  |  |  |  |  |  |  |  |  |
|  | Normal [n/24h] | Baseline | 47 | 8.35 (4.57) | 53 | 7.68 (3.18) | 0.963 |  |  |  |  |  |  |
|  |  | 13 w | 39 | 7.95 (3.81) | 44 | 7.34 (3.22) | 0.763 |  | 18 | 8.63 (4.76) | 43 | 7.41 (3.22) | 0.751 |
|  |  | 26 w | 41 | 8.07 (4.33) | 45 | 7.4 (3.05) | 0.967 |  | 18 | 9.55 (5.57) | 44 | 7.44 (3.08) | 0.43 |
|  |  |  |  |  |  |  |  |  |  |  |  |  |  |
|  | Normal to all ratio | Baseline | 47 | 0.89 (0.15) | 53 | 0.9 (0.15) | 0.854 |  |  |  |  |  |  |
|  |  | 13 w | 39 | 0.88 (0.13) | 44 | 0.88 (0.18) | 0.524 |  | 18 | 0.85 (0.16) | 43 | 0.89 (0.18) | 0.483 |
|  |  | 26 w | 41 | 0.88 (0.14) | 45 | 0.91 (0.13) | 0.54 |  | 18 | 0.85 (0.16) | 44 | 0.91 (0.13) | 0.315 |
|  |  |  |  |  |  |  |  |  |  |  |  |  |  |
|  | Square and/or Dual wave [n/24h] | Baseline | 47 | 1.07 (1.47) | 53 | 0.79 (1.09) | 0.662 |  |  |  |  |  |  |
|  |  | 13 w | 39 | 1.15 (1.4) | 44 | 0.86 (1.17) | 0.382 |  | 18 | 1.56 (1.64) | 43 | 0.8 (1.13) | 0.208 |
|  |  | 26 w | 41 | 1.18 (1.43) | 45 | 0.69 (0.86) | 0.31 |  | 18 | 1.6 (1.64) | 44 | 0.68 (0.87) | 0.115 |
|  |  |  |  |  |  |  |  |  |  |  |  |  |  |
|  | Square and/or Dual wave to all ratio | Baseline | 47 | 0.11 (0.15) | 53 | 0.1 (0.15) | 0.854 |  |  |  |  |  |  |
|  |  | 13 w | 39 | 0.12 (0.13) | 44 | 0.12 (0.18) | 0.524 |  | 18 | 0.15 (0.16) | 43 | 0.11 (0.18) | 0.483 |
|  |  | 26 w | 41 | 0.12 (0.14) | 45 | 0.09 (0.13) | 0.54 |  | 18 | 0.15 (0.16) | 44 | 0.09 (0.13) | 0.315 |
|  |  |  |  |  |  |  |  |  |  |  |  |  |  |
| **Hypoglycemic events** ^b^ | | |  |  |  |  |  |  |  |  |  |  |  |
|  | Glycemia < 50 mg/dl [episodes/pers/24h] | Baseline | 39 | 0.19 (0.2) | 42 | 0.16 (0.3) | 0.321 |  |  |  |  |  |  |
|  |  | 13 w | 38 | 0.14 (0.2) | 42 | 0.17 (0.2) | 0.911 |  | 19 | 0.09 (0.1) | 42 | 0.17 (0.2) | 0.59 |
|  |  | 26 w | 40 | 0.16 (0.3) | 38 | 0.22 (0.3) | 0.148 |  | 18 | 0.11 (0.2) | 38 | 0.22 (0.3) | 0.076 |
|  |  |  |  |  |  |  |  |  |  |  |  |  |  |
|  | Glycemia < 70 mg/dl  [episodes/pers/24h] | Baseline | 39 | 0.85 (0.5) | 42 | 0.67 (0.4) | 0.102 |  |  |  |  |  |  |
|  |  | 13 w | 38 | 0.76 (0.4) | 42 | 0.77 (0.6) | 0.538 |  | 19 | 0.71 (0.4) | 42 | 0.77 (0.6) | 0.802 |
|  |  | 26 w | 40 | 0.74 (0.5) | 38 | 0.98 (0.9) | 0.235 |  | 18 | 0.69 (0.4) | 38 | 0.98 (0.9) | 0.189 |
|  |  |  |  |  |  |  |  |  |  |  |  |  |  |
| **BMI–SDS** | | Baseline | 53 | 0.8 (0.8) | 53 | 0.6 (1) | 0.351 |  |  |  |  |  |  |
|  |  | 13 w | 49 | 0.8 (0.8) | 51 | 0.6 (1) | 0.401 |  | 23 | 0.6 (0.8) | 50 | 0.6 (1) | 0.843 |
|  |  | 26 w | 52 | 0.8 (0.9) | 53 | 0.7 (0.9) | 0.492 |  | 22 | 0.7 (1) | 52 | 0.7 (1) | 0.731 |

Intention-to-treat analysis concerns all available cases. Per protocol analysis concerns patients, who received allocated intervention and declared ELKa system usage for more than 50% of meals. Units are listed in square brackets in the first column.

n, number of patients included to analysis; w, weeks; pers, person; BMI–SDS, Body Mass Index–Standard Deviation Score.

^a^ Data downloaded from insulin pumps. ^b^ Values presented as mean number of episodes per person, per day; based on data downloaded from blood glucose meters, covering 14 days before visit.
